# Antimicrobial activity and cytotoxicity of the ethanol extract, fractions and eight compounds isolated from *Eriosema robustum* (Fabaceae)

**DOI:** 10.1186/1472-6882-13-289

**Published:** 2013-10-29

**Authors:** Maurice D Awouafack, Lyndy J McGaw, Sebastian Gottfried, Roukayatou Mbouangouere, Pierre Tane, Michael Spiteller, Jacobus N Eloff

**Affiliations:** 1 Phytomedicine Programme, Department of Paraclinical Sciences, Faculty of Veterinary Science, University of Pretoria, Private Bag X04, Onderstepoort 0110, South Africa; 2 Laboratory of Natural Products Chemistry, Department of Chemistry, Faculty of Science, University of Dschang, P.O. Box 67, Dschang, Cameroon; 3 Institut für Umweltforschung (INFU) der Fakultät Chemie, Lehrstuhl für Umweltchemie und Analytische Chemie, Technische Universität Dortmund, Otto-Hahn-Strasse 6, D-44221 Dortmund, Germany

**Keywords:** *Eriosema robustum*, Fabaceae, Flavonoids, Antimicrobial, Cytotoxicity

## Abstract

**Background:**

The aim of this study was to evaluate the antimicrobial activity and the cytotoxicity of the ethanol crude extract, fractions and isolated compounds from the twigs of *Eriosema robustum*, a plant used for the treatment of coughs and skin diseases.

**Methods:**

Column chromatographic and spectroscopic techniques were used to isolate and identify eight compounds, robusflavones A (**1**) and B (**2**), orostachyscerebroside A (**3**), stigmasterol (**4**), 1-*O*-heptatriacontanoyl glycerol (**5**), eicosanoic acid (**6**), 3-*O*-β-D-glucopyranoside of sitosterol (**7**) and 6-prenylpinocembrin (**8**), from *E. robustum*. A two-fold serial microdilution method was used to determine the minimum inhibitory concentration (MIC) against fungi and bacteria, and the 3-(4,5-dimethylthiazolyl-2)-2,5-diphenyltetrazolium bromide reduction assay was used to evaluate the cytotoxicity.

**Results:**

Fraction B had significant antimicrobial activity against *Aspergillus fumigatus* and *Cryptoccocus neoformans* (MIC 0.08 mg/ml), whilst the crude extract and fraction A had moderate activity against *A. fumigatus* and *Candida albicans* (MIC 0.16 mg/ml). Fraction A however had excellent activity against *Staphylococcus aureus* (MIC 0.02 mg/ml), *Enterococcus faecalis* and *Escherichia coli* (MIC 0.04 mg/ml). The crude extract had significant activity against *S. aureus*, *E. faecalis* and *E. coli*. Fraction B had good activity against *E. faecalis* and *E. coli* (MIC 0.08 mg/ml). All the isolated compounds had a relatively weak antimicrobial activity. An MIC of 65 μg/ml was obtained with robusflavones A (**1**) and B (**2**) against *C. albicans* and *A. fumigatus*, orostachyscerebroside A (**3**) against *A. fumigatus*, and robusflavone B (**2**) against *C. neoformans*. Compound **8** had the best activity against bacteria (average MIC 55 μg/ml). The 3 fractions and isolated compounds had LC_50_ values between 13.20 to > 100 μg/ml against Vero cells yielding selectivity indices between 0.01 and 1.58.

**Conclusion:**

The isolated compounds generally had a much lower activity than expected based on the activity of the fractions from which they were isolated. This may be the result of synergism between different compounds in the complex extracts or fractions. The results support the traditional use of *E. robustum* to treat infections. The crude extract had a good activity and low preparation cost, and may be useful in topical applications to combat microbial infections.

## Background

The need for new, effective and affordable drugs to treat microbial diseases in the developing world is one of the issues facing global health today. Available drugs for the treatment of these diseases are limited by factors ranging from microbe resistance to safety, compliance and cost. The use of medicinal plants for curative purposes is as old as mankind and can be traced from the beginning of civilisation. The interaction between man and his environment is such that his food, shelter, the diseases that afflict him and the cure of such diseases are all within the environment. Thus, man uses his environment and the resources of nature to combat diseases that afflict him. Consequently over many centuries, people of various countries of the world have embarked on what is now known as traditional medicine [1]. It is estimated that plants are still a major source of primary healthcare for more than 60% of the world’s population.

*Eriosema robustum*, a member of Fabaceae family, is a perennial non-climbing shrub with yellow flowers native to Burundi, Ethiopia, Kenya, Rwanda, Tanzania, Uganda, Democratic Republic of Congo and Cameroon [2]. It is used traditionally for the treatment of coughs in East Africa [3] and skin diseases in the Western Region of Cameroon. Previous phytochemical study on this species led to the isolation and characterization of two flavonoids, robusflavones A and B, and five known compounds [4]. As part of our ongoing study for bioactive secondary metabolites from species of the Fabaceae family [4-6], we have re-investigated the ethanol extract of the twigs of *E. robustum* using bioassay-guided fractionation. To investigate the potential use of an extract, fractions or compounds, the cellular toxicity also has to be determined. The therapeutic index, that is, ratio of activity against microorganisms to toxicity to animal cells, is important.

We report herein the antimicrobial activity and cytotoxicity of the crude extract, fractions and the isolated compounds (**1**–**8**). This is also the first report on the isolation of a cerebroside, orostachyscerebroside A (**3**), from the genus *Eriosema*.

## Methods

### Plant material

The twigs of *E. robustum* were collected in Dschang, Western Region of Cameroon, in December 2011 and identified at the Cameroon National Herbarium in Yaoundé where a specimen was deposited under a voucher number 35291/HNC.

### Extraction and isolation

The dried and powdered twigs of *E. robustum* (2 kg) were extracted for three days in ethanol (10 litres × 3 times) to yield the crude extract (115 g) after filtration and solvent evaporation using a rotary evaporator. Part of this extract (5 g) was subjected to a silica gel column chromatography eluted with n-hexane (Hex), chloroform (CHCl_3_), ethyl acetate (EtOAc) and methanol (MeOH) in increasing polarity to give 40 fractions of 500 ml each that were combined after monitoring with comparative thin layer chromatography (Co-TLC) into 3 fractions: A [(0.9 g, Hex - CHCl_3_ (100:0, 4:1, 3:2, 1:4) and CHCl_3_ - EtOAc (100:0, 4:1)], B [(0.7 g, CHCl_3_ - EtOAc (4:1, 3:2, 1:4) and C [(2.5 g, CHCl_3_ – EtOAc (1:4) and EtOAc - MeOH (100:0, 3:7, 0:100)]. Fraction C did not have as many active antimicrobial constituents separated by TLC in bioautography as fractions A and B, and was not further investigated.

Fraction B was subjected to purification applying silica gel column chromatography eluted with n-hexane, acetone and methanol in order of increasing polarity to yield 50 fractions of 150 ml each which were combined in subfractions after monitoring with Co-TLC. Subfractions F_25-26_ and F_27-30_ eluted with n-hexane: acetone (17:3) gave compounds **2** (9 mg), **4** (8 mg) and **6** (12 mg). Subfractions F_20-26_ eluted with n-hexane: acetone (7:3, 3:2) and F_27-35_ eluted with n-hexane: acetone (11:9, 1:9) were similarly subjected to further silica gel column chromatography eluted with n-hexane, ethyl acetate, methanol in gradient polarity followed by preparative TLC and Sephadex LH-20 to yield **1** (4 mg), **3** (10 mg), **5** (7 mg) and **7** (9 mg).

Fraction A was subjected to Sephadex LH-20 to remove chlorophyll and the eluate was concentrated and fractionated using similar silica gel column techniques as described above for fraction B to give mainly **4** (3 mg) and **8** (17 mg).

*Orostachyscerebroside A* (**3**): colourless amorphous; ^1^H NMR (DMSO-*d*_*6*_, 400 MHz): *δ* 4.38 (br.*t*, 4.0 Hz, H-1_b_), 4.58 (br.*t*, 4.0 Hz, H-1_a_), 4.08 (*m*, H-2), 3.76 (*m*, H-3), 3.70 (*m*, H-4), 1.86-2.02 (2H, *m*, H-5), 5.63 (8H, *m*, H-6/H-7/H-10/H-11/H-6′/H-7′/H-9′/H-10′), 1.23-1.33 (8H, *m*, H-8/H-12/H-5′/H-11′), 1.40-1.65 (2H, *m*, H-9), 1.24-1.31 (20H, *m*, H-13 to H-22), 1.50 (4H, *m*, H-23/H-19′), 0.84 (6H, *t*, 4.0 Hz, H-24/H-20′), 3.80 (*m*, H-2′), 1.26 (*m*, H-3′_a_), 2.02 (*m*, H-3′_b_), 1.02 (2H, *m*, H-4′), 1.02-1.20 (2H, *m*, H-8′), 1.20-1.33 (14H, *m*, H-12′ to H-18′), 4.12 (*d*, 8.0 Hz, Glc-1″), 3.32 (*m*, H-2″), 4.96 (*m*, H-3″), 3.60 (*m*, H-4″), 3.03 (*m*, H-5″), 3.38 (*m*, H-6″_a_), 3.65 (*m*, H-6″_b_), 7.55 (*d*, 8.0 Hz, NH); ^13^C NMR (DMSO-*d*_*6*_, 100 MHz) *δ*: 69.4 (C-1), 50.3 (C-2), 70.9 (C-3), 71.3 (C-4), 34.8 (C-5), 129.8 (C-6), 130.1 (C-7), 27.4 (C-8/C-11′), 32.2 (C-9), 130.3 (C-10), 130.7 (C-11), 31.8 (C-12), 29.0-30.9 (C-13 to C-22), 22.6 (C-23/C-19′), 14.4 (Me-24/Me-20′), 174.2 (C-1′), 71.3 (C-2′), 34.8 (C-3′), 24.4 (C-4′/C-8′), 27.1 (C-5′), 130.1 (C-6′/C-10′), 129.8 (C-7′/C-9′), 29.0-30.9 (C-12′ to C-18′), 103.9 (C-1″), 73.9 (C-2″), 76.9 (C-3″), 70.4 (C-4″), 77.3 (C-5″), 61.4 (C-6″); MALDI-FT-MS *m/z*: 866.6720 ([M + H]^+^, C_50_H_92_O_10_N), MALDI-FT-MS^2^ (CID at 45 eV) (rel. int.) *m/z*: 704.6171 [(M + H)-162, C_44_H_82_NO_5_, 51]^+^, 686.6065 [(M + H)-162-H_2_O, C_44_H_80_NO_4_, 70]^+^, 500.3176 [(M + H)-366, C_26_H_46_NO_8_, 100]^+^.

### General experimental procedures

IR spectra were recorded on a Bruker Alpha FT-IR spectrometer (Optik GmbH, Germany). UV spectra were recorded on Thermo Electron Helios spectrophotometer (UVB 120726, England).

^1^H- and ^13^C-NMR spectra were recorded with a Bruker spectrometer at 500 MHz and a Varian spectrometer at 400 MHz. Chemical shifts (*δ*) were quoted in parts per million (ppm) from the internal standard tetramethylsilane (TMS). Deuterated solvents as dimethyl sulfoxide (DMSO-*d*_*6*_, for compounds **1**, **2**, **3**, **7** and **8**), and chloroform (CDCl_3_, for compounds **4**, **5** and **6**) were used for the NMR experiments. Mass spectrometry was performed on a Thermo Scientific LTQ-Orbitrap instrument coupled to a MALDI (matrix-assisted laser desorption ionization) or liquid chromatography electrospray ionization source. Column chromatography was performed on MN silica gel 60 (0.063-0.2 mm/70–230) mesh. Preparative TLC was performed using high-purity grade silica gel powder (60 A, 2–25 μm) Sigma-Aldrich, Germany. Pre-coated plates of TLC silica gel 60 F_254_ (Merck, Germany) were used for monitoring fractions and spots were detected with UV light (254 and 365 nm) and then sprayed with 30% H_2_SO_4_ followed by heating up to 110°C.

### Antimicrobial assay

The two-fold serial microdilution method described by Eloff (1998) was used to determine the minimum inhibitory concentration (MIC) values for the extract, fractions and isolated compounds against bacteria [7] and a modification of [7] by Masoko *et al*. (2005) was used for fungi [8]. The respective MIC of the samples was determined using two Gram-positive bacteria, *Staphylococcus aureus* (ATCC 29213) and *Enterococcus faecalis* (ATCC 29212), two Gram-negative bacteria, *Pseudomonas aeruginosa* (ATCC 27853) and *Escherichia coli* (ATCC 25922); and three clinical isolates of the pathogenic fungi *Candida albicans*, *Cryptococcus neoformans* and, *Aspergillus niger* along with *Candida albicans* (ATCC 10231). Some fungal strains used were cultured from clinical cases of fungal infectious diseases in animals (before treatment) in the Department of Veterinary Tropical Diseases, Faculty of Veterinary Science. *C. albicans* was isolated from a Gouldian finch, *C. neoformans* from a cheetah, while *A. fumigatus* was isolated from a chicken which suffered from a systemic mycosis.

In the antibacterial tests, 100 μl of the (10 mg/ml) crude extract and fractions or (1 mg/ml) compounds in duplicate dissolved in dimethyl sulfoxide (DMSO) were serially diluted two-fold with sterile distilled water in 96-well microtitre plates and 100 μl bacterial culture in Mueller Hinton broth (MHB) (Fluka, Germany) was added to each well. DMSO was used to dissolve the compounds because acetone used in the original method [7] did not dissolve all the compounds well. The densities of bacterial cultures were approximately 2.6 × 10^12^ cfu/ml*, S. aureus*; 1.5 × 10^10^ cfu/ml, *E. faecalis*; 5.2 × 10^13^ cfu/ml, *P. aeruginosa* and 3.0 × 10^11^ cfu/ml*, E. coli*, obtained by adding 99 ml of MHB to 1 ml of each microorganism from the freshly prepared overnight bacterial culture in Mueller Hinton broth [9]. Gentamicin and DMSO were used as positive and negative controls, respectively. The microtitre plates were sealed in plastic bags to avoid contamination in the laboratory and were incubated overnight at 37°C. Afterwards, 40 μl of 0.2 mg/ml of *p*-iodonitrotetrazolium violet (INT) (Sigma, Germany) was added to each well to indicate microbial growth. The microtitre plates were further incubated at 37°C for 2 h and the minimal inhibitory concentration (MIC) was determined as the lowest concentration inhibiting microbial growth, indicated by a decrease in the intensity of the red colour of the formazan [10].

For the antifungal assay, 100 μl of the crude extract, fractions and compounds dissolved in DMSO at the same concentrations, as described above for the bacteria, were two-fold serially diluted with distilled water in 96-well microtitre plates, and 100 μl of fungal culture in Potato Dextrose broth (Sigma, Germany) was added to each well [8]. Amphotericin B and DMSO were used as positive and negative controls, respectively. INT (40 μl) at 0.2 mg/ml in distilled water was added to each well as a growth indicator and the covered microplates were incubated at 35°C after sealing in a plastic bag. The MIC value was recorded after 16 and 24 h incubation for *C. albicans* and *A. fumigatus*, and after 24 and 36 h incubation for *C. neoformans* as the lowest concentration of the sample that inhibited fungal growth. The colourless salt of tetrazolium acts as an electron acceptor and is reduced to a red coloured formazan product by biologically active organisms [11,12]. The solution in the well remains clear or shows a marked decrease in intensity of colour after incubation with INT at the concentration where fungal growth is inhibited. The experiment was performed in triplicate and the standard deviation was zero.

### Cytotoxicity assay

The MTT (3-(4,5-dimethylthiazolyl-2)-2,5-diphenyltetrazolium bromide) reduction assay is one of the most frequently used methods for measuring cell proliferation and cytotoxicity. The intensity of colour (measured spectrophotometrically) of the MTT formazan produced by living, metabolically active cells is proportional to the number of live cells present. MTT is a yellow water-soluble tetrazolium dye that is reduced by live, but not dead, cells to a purple formazan product that is insoluble in aqueous solutions. The viable cell growth after incubation with extract, fractions and isolated compounds was determined using the tetrazolium-based colorimetric MTT assay described by Mosmann (1983) [13]. In short, monkey Vero cells of a subconfluent culture were harvested and centrifuged at 200 × *g* for 5 min, and resuspended in growth medium to 5 × 10^4^ cells/ml. The growth medium used was Minimal Essential Medium (MEM, Sigma) supplemented with 0.1% gentamicin (Virbac) and 5% foetal calf serum (Sigma). A total of 200 μl of the cell suspension is pipetted into each well of columns 2 to 11 of a sterile 96-well microtitre plate. A 200 μl aliquot of growth medium was added to wells of columns 1 and 12 to minimize the “edge effect” and maintain humidity. The plates were incubated for 24 h at 37°C in a 5% CO_2_ incubator, until the cells were in the exponential phase of growth. The MEM was aspirated from the cells, and replaced with 200 μl of extract, fractions and compounds at differing concentrations (serial dilution prepared in growth medium). The cells were disturbed as little as possible during the aspiration of medium and addition of test samples. Each dilution was tested in quadruplicate. The microtitre plates were incubated at 37°C in a 5% CO_2_ incubator for 48 h with test compounds, fractions or extract. Untreated cells and positive control (doxorubicin, Pfizer Laboratories) were included. After incubation, cells were washed with 150 μl phosphate buffered saline (PBS) and fresh MEM (200 μl) was added to each well together with 30 μl MTT (Sigma, stock solution of 5 mg/ml in PBS). The plates were then incubated for a further 4 h at 37°C. After incubation with MTT the medium in each well was carefully removed, without disturbing the MTT crystals in the wells. The MTT formazan crystals were dissolved by adding 50 μl DMSO to each well and the plates gently shaken until the MTT solution was dissolved. The amount of MTT reduction was measured immediately by detecting absorbance in a microplate reader (Versamax, Molecular Devices) at a wavelength of 570 nm and a reference wavelength of 630 nm. The wells in column 1, containing medium and MTT but no cells, were used to blank the plate reader. The LC_50_ values were calculated as the concentration of test sample resulting in a 50% reduction of absorbance compared to untreated cells.

## Results and discussion

### Chemical composition and antimicrobial activity

**Figure 1:**
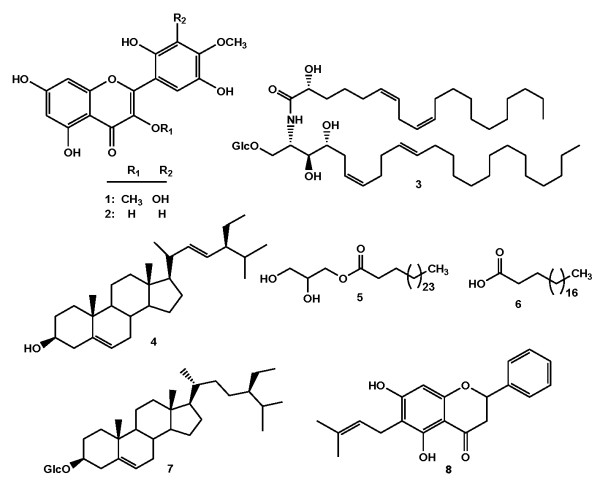
**Chemical structures of compounds (1–8) isolated from *E. robustum.***

The EtOH crude extract from the twigs of *Eriosema robustum* was subjected to repeated silica gel column chromatography followed by preparative TLC and Sephadex LH-20 to afford eight compounds including robusflavones A (**1**, yellow amorphous, *m/z* 362, C_17_H_14_O_9_) and B (**2**, yellow amorphous, *m/z* 332, C_16_H_12_O_8_) [4], orostachyscerebroside A (**3**) [14], stigmasterol (**4**, white powder, *m/z* 412, C_29_H_48_O) [15], 1-*O*-heptatriacontanoyl glycerol (**5**, white powder, *m/z* 484, C_30_H_60_O_4_) [16], eicosanoic acid (**6**, white powder, *m/z* 312, C_20_H_40_O_2_) [17], 3-*O*-β-D-glucopyranoside of sitosterol (**7**, white crystals, *m/z* C_35_H_60_O_6_) [18] and 6-prenylpinocembrin (**8**, yellow, *m/z* C_20_H_20_O_4_) [19] (Figure 1). The structures elucidation of the compounds were achieved by interpretation of their NMR, IR, UV and MS data, and by comparison with those reported in the literature. To the best of our knowledge this is the first report on the isolation of a cerebroside, orostachyscerebroside A (**3**), from the genus *Eriosema*. Orostachyscerebroside A (**3**) was found for the first time in the aerial parts of *Orostachys japonicas* in 2012 [14].

The antimicrobial activity and cytotoxicity of the crude extract, fractions from the column chromatography of the extract and isolated compounds (**1**–**8**) were determined and the results are presented in Tables 1, 2 and 3. Many authors consider the antimicrobial activity of extracts to be significant if the MIC value is 0.1 mg/ml or lower, moderate if 0.1 < MIC ≤ 0.625 mg/ml and weak if MIC > 0.625 mg/ml [20,21]. When reporting the antimicrobial activity of the isolated compounds, it is significant if the MIC is 10 μg/ml or lower, moderate if 10 < MIC ≤ 100 μg/ml and low if MIC > 100 μg/ml [21,22]. Based on these criteria, all the tested samples had significant to weak antifungal activities against the four fungi with MIC values between 80 and 1250 μg/ml (Table 1). Fraction B was significantly active against *Aspergillus fumigatus* and *Cryptoccocus neoformans* with an MIC of 0.08 mg/ml, while the crude extract and fraction A had moderate activity against *Aspergillus fumigatus* and *Candida albicans* (ATCC) with an MIC value of 0.16 mg/ml in all cases. Fraction B had the highest antifungal activity among the tested fractions and extract with an average MIC of 0.61 mg/ml followed by fraction A (average MIC of 0.65 mg/ml) and the crude extract (average MIC of 0.88 mg/ml).

**Table 1:** **Minimum inhibitory concentration (MIC) of the extract, fractions and compounds (1–8) from *E. robustum *** **against fungi (C.a, C.A, A.f, C.n) and bacteria (S.a, P.a, E.f, E.c)**

**Samples**	**Fungi (MIC values)**									**Bacteria (MIC values)**				
	**C.a**		**C.A**		**A.f**		**C.n**^**a**^		**Average**	**S.a**	**P.a**	**E.f**	**E.c**	**Average**
	**16 h**	**24 h**	**16 h**	**24 h**	**16 h**	**24 h**	**24 h**	**36 h**						
**Extract and Fractions (mg/ml)**														
Crude extract	1.25	1.25	1.25	1.25	0.16	0.63	0.63	0.63	0.88	0.08	0.31	0.08	0.08	0.14
Fraction A	0.63	0.63	0.16	0.32	0.32	0.63	1.25	1.25	0.65	0.02	0.31	0.04	0.04	0.10
Fraction B	1.25	1.25	0.32	0.63	0.08	0.63	0.08	0.63	0.61	0.16	0.31	0.08	0.08	0.16
**Compounds (μg/ml)**														
Robusflavone A (**1**)	250	250	65	65	65	125	250	250	165	250	63	250	250	203.3
Robusflavone B (**2**)	125	125	65	65	65	125	65	65	88	125	63	63	125	94
Orostachyscerebroside A (**3**)	250	250	130	250	130	250	250	250	220	125	63	125	125	109.5
Stigmasterol (**4**)	125	125	125	125	65	65	125	125	110	125	63	250	250	172
1-*O*-Heptatriacontanoyl glycerol (**5**)	250	250	65	65	125	125	250	250	173	125	63	125	125	109.5
Eicosanoic acid (**6**)	250	250	125	125	125	125	> 250	> 250	188	63	63	125	125	94
3-*O*-β-D-Glucopyranoside of sitosterol (**7**)	125	125	125	125	65	125	65	125	110	125	63	63	125	94
6-Prenylpinocembrin (**8**)	125	125	65	65	125	250	> 250	> 250	157	31.3	63	63	63	55.1
**Controls (μg/ml)**														
Amp B	30	30	10	30	65	125	> 250	> 250	99	-	-	-	-	-
Gen	-	-	-	-	-	-	-	-	-	< 3.91	< 3.91	< 3.91	< 3.91	< 3.91
DMSO^b^	na	na	na	na	na	na	na	na		na	na	na	na	

C.a: *Candida albicans* (isolate), C.A: *Candida albicans* (ATCC), C.n: *Cryptococcus neoformans,* A.f: *Aspergillus fumigatus,* E.c: *Escherichia coli,* E.f: *Enterococcus faecalis,* S.a: *Staphylococcus aureus,* P.a: *Pseudomonas aeruginosa,* Amp B: Amphotericin B, Gen: Gentamicin, ^a^With this microorganism little reaction was observed after 16 h and values were measured after 24 and 36 h, ^b^The final concentration of DMSO used was less than 10%, na: not active.

The results are the means of three replicates for fungi and two replicates for bacteria and the standard deviation was zero.

**Table 2:** **Total activity in ml/g of crude extract and fractions (A and B) from *E. robustum *** **calculated by dividing mass in g of extract or fractions with their MIC values**[20]

**Samples**	**Fungi**									**Bacteria**				
	**C.a**		**C.A**		**A.f**		**C.n**		**Average**	**S.a**	**P.a**	**E.f**	**E.c**	**Average**
	**16 h**	**24 h**	**16 h**	**24 h**	**16 h**	**24 h**	**24 h**	**36 h**						
**Extract and Fractions**														
Crude extract	4000	4000	4000	4000	31250	7937	7937	7937	8883	62500	16129	62500	62500	50907
Fraction A	1429	1429	5625	2813	2813	1429	720	720	2123	45000	2903	22500	22500	23226
Fraction B	560	560	2188	1111	8750	1111	8750	1111	3018	4375	2258	8750	8750	6033
**Compounds**														
Robusflavone A (**1**)	4	4	13.4	13.4	13.4	8	4	4	8	4	16	4	4	7
Robusflavone B (**2**)	8	8	13.4	13.4	13.4	8	13.4	13.4	11	8	16	16	8	12
Orostachyscerebroside A (**3**)	4	4	7.7	4	7.7	4	4	4	4.5	8	16	8	8	9.1
Stigmasterol (**4**)	8	8	8	8	13.4	13.4	8	8	9	8	16	4	4	8
1-*O*-Heptatriacontanoyl glycerol (**5**)	4	4	13.4	13.4	8	8	4	4	7	8	16	8	8	10
Eicosanoic acid (**6**)	4	4	8	8	8	8	< 8	< 8	7	16	16	8	8	12
3-*O*-β-D-Glucopyranoside of sitosterol (**7**)	8	8	8	8	13.4	8	13.4	8	9	8	16	16	8	12
6-Prenylpinocembrin (**8**)	8	8	13.4	13.4	8	4	< 4	< 4	8	32	16	16	16	20

C.a: *Candida albicans* (isolate), C.A: *Candida albicans* (ATCC), C.n: *Cryptococcus neoformans,* A.f: *Aspergillus fumigatus,* E.c: *Escherichia coli,* E.f: *Enterococcus faecalis,* S.a: *Staphylococcus aureus,* P.a: *Pseudomonas aeruginosa*.

The inverse of MIC in ml/mg was calculated for compounds (**1–8**).

**Table 3:** **Cytotoxicity of the crude extract, fractions and compounds from *E. robustum *** **and their selectivity index (SI)**

**Samples**	**Cytotoxicty (LC**_**50**_**, μg/ml)**	**Selectivity index**^**a**^							
		**C.a**	**C.A**	**A.f**	**C.n**	**S.a**	**P.a**	**E.f**	**E.c**
**Extract and fractions**									
Crude extract	53.45 ± 2.21	0.04	0.04	0.21*	0.08	0.67	0.17	0.67	0.67
Fraction A	17.91 ± 1.71	0.03	0.09*	0.05*	0.01	0.90	0.06	0.45	0.45
Fraction B	93.76 ± 25.62	0.08	0.22*	0.66*	0.66*	0.59	0.30	1.17	1.17
**Compounds**									
Robusflavone A (**1**)	13.20 ± 0.02	0.05	0.20	0.16*	0.05	0.05	0.21	0.05	0.05
Robusflavone B (**2**)	30.42 ± 3.26	0.24	0.47	0.36*	0.47	0.24	0.48	0.48	0.24
Orostachyscerebroside A (**3**)	> 100	0.4	0.53*	0.53*	0.4	0.8	1.59	0.8	0.8
Stigmasterol (**4**)	54.97 ± 11.05	0.44	0.44	0.85	0.44	0.44	0.87	0.22	0.22
1-*O*-Heptatriacontanoylglycerol (**5**)	91.52 ± 28.38	0.37	1.41	0.73	0.37	0.73	1.45	0.73	0.73
Eicosanoic acid (**6**)	NT	-	-	-	-	-	-	-	-
3-*O*-β-D-Glucopyranoside of sitosterol (**7**)	>100	0.80	0.80	1.17*	1.17*	0.80	1.58	1.58	0.80
6-Prenylpinocembrin (**8**)	21.87 ± 0.37	0.17	0.34	0.13*	0.09	0.70	0.35	0.35	0.35
**Control**									
Dox	3.32 ± 1.31								

C.a: *Candida albicans* (isolate), C.A: *Candida albicans* (ATCC), A.f: *Apergillus fumigatus*, C.n: *Cryptoccocus neoformans,* E.c: *Escherichia coli,* E.f: *Enterococcus faecalis,* S.a: *Staphylococcus aureus,* P.a: *s* Dox: Doxorubicin with LC_50_ in μM, NT: not tested, ^a^SI = LC_50_ /MIC, *SI obtained from average MIC.

The most active isolated compounds had moderate antifungal activity with the MIC value of 65 μg/ml. These included robusflavones A (**1**) and B (**2**) against *C. albicans* (ATCC) and *A. fumigatus*, compounds **4** and **7** against *C. albicans* (ATCC), as well as compound **6** against *A. fumigatus* and *C.neoformans,* orostachyscerebroside A (**3**) against *A. fumigatus*, and robusflavone B (**2**) against *C. neoformans*. Compounds **5** and **7** had weak antifungal activity against *C. neoformans* (MIC > 250 μg/ml in both cases). The animal pathogenic isolate *C. albicans* was more resistant to the tested samples with MIC values between 0.63 to 1.25 mg/ml for the extract and fractions and ranging from 125 to 250 μg/ml for compounds, respectively. Nevertheless, robusflavone B (**2**) had the best antifungal activity (average MIC of 88 μg/ml), followed by compounds **3** and **6** (average MIC of 110 μg/ml), compound **7** (average MIC of 157 μg/ml) and robusflavone A (**1**) (average MIC of 165 μg/ml).

The antibacterial activity of the crude extract, fractions and isolated compounds were determined against four bacteria with MIC values ranging from 0.02 to 0.31 mg/ml and from 31.3 to 250 μg/ml, respectively (Table 1). The crude extract had good activity against *S. aureus*, *E. faecalis* and *E. coli* (MIC value of 0.08 mg/ml) and had moderate activity against *P. aeruginosa* (MIC value of 0.31 mg/ml). Fraction A had excellent activity against *S. aureus* (MIC of 0.02 mg/ml), *E. faecalis* and *E. coli* (MIC of 0.04 mg/ml) and reasonable activity against *P. aeruginosa* (MIC value of 0.31 mg/ml). The Gram-negative *P. aeruginosa* and the Gram-positive *S. aureus* were more resistant to fraction B with MIC values of 0.31 and 0.16 mg/ml, respectively. However, fraction B had significant activity against *E. faecalis* and *E. coli* with an MIC value of 0.08 mg/ml. Compound **8** had moderate activity against *S. aureus* (MIC value of 31.3 μg/ml) and had reasonable activity against other bacteria with an MIC value of 63 μg/ml. Other constituents had also reasonable antibacterial activity against *P. aeruginosa* with the MIC value of 63 μg/ml as well as robusflavone B (**2**) and compound **6** against *E. faecalis* (MIC of 63 μg/ml). Amongst all the isolated compounds, 6-prenylpinocembrin (**8**) had the highest antibacterial activity with an average MIC of 55.1 μg/ml and average total activity of 20 ml/mg followed by robusflavone B (**2**), and compounds **5** and **6** (average MIC of 94 μg/ml and average total activity of 12 ml/mg), as well as robusflavone A (**1**) with an average MIC of 203.3 μg/ml and average total activity of 7 ml/mg.

The crude extract and fractions were found to be more active than the isolated compounds against the bacteria and the fungi. The antibacterial activity of fraction A is noteworthy against the bacteria with an average MIC value of 100 μg/ml [20,23] and an average total activity of 23 226 ml/g. Fraction B had good antifungal activity against *A. fumigatus* and *C. neoformans* with an MIC value of 80 μg/ml and total activity of 8 750 ml/g after 16 and 24 h of incubation. This implies that if 1 g of fraction B from the ethanol extract was diluted to 8.75 litres it would still inhibit the growth of *A. fumigatus* and *C. neoformans*. Compound **8**, obtained from the most antibacterial-active part, fraction A, had the highest antibacterial activity amongst all the isolated compounds with an average MIC of 55.1 μg/ml and average total activity of 20 ml/mg. The antibacterial activity of compound **8** is in agreement with previous results reported on the antimicrobial activity of similar compounds bearing the prenyl group on their structures [6,24]. Robusflavones A (**1**) and B (**2**) characterized earlier as two new antioxidant flavones [4] had moderate activities against fungi and bacteria with mean MIC values of 165, 88, 203.3 and 94 μg/ml, and average total activities of 8, 11, 7 and 12 ml/mg, respectively. The cerebroside and the glycerol derivative (compounds **3** and **5**) had similar antibacterial activity (both with average MIC of 109.5 μg/ml) but had slightly different antifungal activity with average MIC values of 220 and 173 μg/ml, respectively.

From the results, it is evident that the isolated compounds did not have good activity against the tested microorganisms as observed for extract and fractions. This supports the conclusion of Lewis and Ausubel (2006) that plants contain many compounds with low antimicrobial activity and use other mechanisms to combat microbial growth [25]. In some of our previous work, it has been found that crude extracts and fractions have excellent potential activity in treating microbial infections and may even have as good or higher activity than commercially used antimicrobials in the field or clinical trials [26,27]. This could be justified by the fact that, constituents in plant extract or fractions interacting in a synergistic manner may not be highly active when they are not part of a mixture with synergistic compounds. The antimicrobial activity of the crude extract, fractions and compounds (**1**–**3**, **5**, **6** and **8**) are reported here for the first time.

### Cytotoxicity

The crude extract, fractions and isolated compounds from the twigs of *E. robostum* were evaluated for their cytotoxicity against monkey kidney Vero cells *in vitro* using the MTT (3-(4,5-dimethylythiazol-2-yl)-2,5-diphenyl-2H-tetrazolium hydrobromide) assay and the results are presented in Table 3. Apart from eicosanoic acid (**6**) that was not tested, compounds **3** and **7** had LC_50_ value greater than 100 μg/ml whilst the crude extract, fractions and others isolated compounds (**1**, **2**, **4**, **5** and **8**) had cytotoxic activity against the Vero cells with LC_50_ values ranging from 13.20 to 91.52 μg/ml. Our samples, and especially the tested compounds, could be considered less toxic than the positive control doxorubicin (LC_50_ = 3.32 μM) and by taking into consideration the criterion of the American National Cancer Institute (NCI) regarding the cytotoxicity of pure compounds (LC_50_ < 4 μg/ml) [28]. However, the LC_50_ values of 13.20, 30.42 and 21.87 μg/ml for compounds **1**, **2** and **8** with the same basic skeleton (flavonoid) were lower than those of others compounds and more cell lines should be tested in a comprehensive screen to confirm their probable safety in terms of cytotoxicity. The selectivity index (SI) values of the tested samples ranged from 0.01 to 1.58 and could be considered as poor when taking in consideration that the ratio for a good therapeutic index for a remedy or drug should be ≥ 10 [29]. This is the first report on the cytotoxicity of the extract, fractions as well as compounds **1**, **2**, **3**, **5** and **8** from *E. robustum* against Vero cells.

Fraction B had good antifungal activity with a total activity of 3018 ml/g for the fraction. One should keep in mind that the 5 g of extract separated by column chromatography was obtained from 87 g of the original dried plant material. In comparing the activity of different plants the concept of total activity was developed [30]. This indicates the volume to which level the extract from 1 g of dried plant material can be diluted and still inhibit the growth of the microorganism. The total activity of 3018 ml/g for fraction B was derived from 87 g of the original dried plant material. The total activity per gram dried plant material for fungi was therefore c. 35 ml/g. The same argument shows that fraction A had a total antibacterial activity of 267 ml/g dried plant material. The crude extract and fractions had fungistatic effect in most of the cases such as the crude extract against *A. fumigatus*, fraction A against *C. albicans* and *A. fumigatus*, and fraction B against *C. albicans*, *A. fumigatus* and *C. neoformans*. It is noteworthy that the crude extract had a fungicidal activity against the two *Candida* and the *Cryptococcus* species, but the fractions had mainly fungistatic activity. This again points to the probability of synergistic activities.

With the high total activity of the crude extract and the ease of preparation, there is a reasonable possibility that the crude extract can be used as topical treatment for fungal and especially bacterial infections. Manipulation of the extract by for example solvent-solvent fractionation may lead to extracts with a higher activity and possibly a lower cytotoxicity [31].

## Conclusion

The results obtained support the traditional use of *E. robustum* to treat infections. The crude extract had good activity with low toxicity to animal cells, and may be useful in topical applications to combat microbial infections.

## Competing interests

The authors declare that they have no competing interests.

## Authors’ contributions

MDA carried out the phytochemical and antimicrobial studies, and wrote the manuscript; LJM carried out the cytotoxicity and participated in the manuscript writing; SG and RM assisted with the structure elucidation of some compounds; PT, MS and JNE supervised the work. All the authors read and approved the final manuscript after revision by JNE.

## Pre-publication history

The pre-publication history for this paper can be accessed here:

http://www.biomedcentral.com/1472-6882/13/289/prepub

## References

[B1] Ogungbamila O Niemeyer HM Investigation of anti-infective activities of local plant materials used as drugs Results, lessons learned, and prospects for development of sustainable research environments in developing countries 1997 Santiago: IPICS

[B2] Gillett JB Polhill RM Verdcourt B Milne-Redhead E, Polhill RM Papilionoideae Flora of Tropical East Africa, Leguminosae 1971 Part 4 London: Crown Agents for Overseas Governments and Administrations 635 637

[B3] Kokwaro JO Medicinal plants of East Africa 2009 3 Nairobi: University of Nairobi Press 335

[B4] Awouafack MD Tane P Eloff JN Two new antioxidant flavones from the twigs of *Eriosema robustum* (Fabaceae) Phytochemistry Lett 2013 6 62 66 10.1016/j.phytol.2012.10.017

[B5] Awouafack MD Spiteller P Lamshöft M Kusari S Ivanova B Tane P Spiteller M Antimicrobial isopropenyl-dihydrofuranoisoflavones from *Crotalaria lachnophora* J Nat Prod 2011 74 272 278 10.1021/np1005218 21265557

[B6] Awouafack MD Kouam SF Hussain H Ngamga D Tane P Schulz B Green IR Krohn K Antimicrobial prenylated dihydrochalcones from *Eriosema glomerata* Planta Med 2008 74 50 54 10.1055/s-2007-993782 18203062

[B7] Eloff JN A sensitive and quick microplate method to determine the minimal inhibitory concentration of plant extracts for bacteria Planta Med 1998 64 711 713 10.1055/s-2006-957563 9933989

[B8] Masoko P Picard J Eloff JN Antifungal activities of six South African *Terminalia* species (Combretaceae) J Ethnopharmacol 2005 99 301 308 10.1016/j.jep.2005.01.061 15894142

[B9] Suleiman MM McGaw LJ Naidoo V Eloff JN Evaluation of several tree species for activity against the animal fungal pathogen *Aspergillus fumigatus* S Afr J Bot 2010 76 64 71 10.1016/j.sajb.2009.07.001

[B10] Shai LJ McGaw LJ Masoko P Eloff JN Antifungal and antibacterial activity of seven traditionally used South African plant species active against *Candida albicans* S Afr J Bot 2008 74 677 684 10.1016/j.sajb.2008.04.003

[B11] Mahlo SM McGaw LJ Eloff JN Antifungal activity of leaf extracts from South African trees against plant pathogens Crop Prot 2010 29 1529 1533 10.1016/j.cropro.2010.08.015

[B12] Eloff JN Which extractant should be used for the screening and isolation of antimicrobial components from plants? J Ethnopharmacol 1998 60 1 8 10.1016/S0378-8741(97)00123-2 9533426

[B13] Mosmann T Rapid colorimetric assay for cellular growth and survival: application to proliferation and cytotoxicity assays J Immunol Methods 1983 65 55 63 10.1016/0022-1759(83)90303-4 6606682

[B14] Zhang H Oh J Jang T Min BS Na M Glycolipids from the aerial parts of *Orostachys japonicus* with fatty acid synthase inhibitory and cytotoxic activities Food Chem 2012 131 1097 1103 10.1016/j.foodchem.2011.09.058

[B15] Forgo P Kövér KE Gradient enhanced selective experiments in the ^1^H NMR chemical shift assignment of the skeleton and side-chain resonances of stigmasterol, a phytosterol derivative Steroids 2004 69 43 50 10.1016/j.steroids.2003.09.012 14715376

[B16] Qi S-H Zhang S Huang J-S Xiao Z-H Wu J Long L-J Glycerol derivatives and sterols from *Sargassum parvivesiculosum* Chem Pharm Bull 2004 52 986 988 10.1248/cpb.52.986 15304997

[B17] Jain R Sharma P Bhagchandani T Jain SC Phytochemical investigation and antimicrobial activity of *Acacia senegal* root heartwood J Pharm Res 2012 5 4934 4938

[B18] Al-Oqail M Hassan WHB Ahmad MS Al-Rehaily AJ Phytochemical and biological studies of *Solanum schimperianum* Hochst Saudi Pharm J 2012 20 371 379 10.1016/j.jsps.2012.05.010 23960812 PMC3744960

[B19] Caffaratti M Ortega MG Scarafia ME Ariza Espinar L Juliani HR Prenylated flavanones from *Dalea elegans* Phytochemistry 1994 36 1082 1084 10.1016/S0031-9422(00)90498-9

[B20] Eloff JN Quantification the bioactivity of plant extracts during screening and bioassay guided fractionation Phytomedicine 2004 11 370 371 10.1078/0944711041495218 15185853

[B21] Kuete V Potential of Cameroonian plants and derived products against microbial infections: a review Planta Med 2010 76 1479 1491 10.1055/s-0030-1250027 20533165

[B22] Ríos JL Recio MC Medicinal plants and antimicrobial activity J Ethnopharmacol 2005 100 80 84 10.1016/j.jep.2005.04.025 15964727

[B23] Kuete V Ango PY Fotso GW Kapche GDWF Dzoyem JP Wouking AG Ngadjui BT Abegaz BM Antimicrobial activities of the methanol extract and compounds from *Artocarpus communis* (Moraceae) BMC Complement Altern Med 2011 11 42 10.1186/1472-6882-11-42 21612612 PMC3118951

[B24] Awouafack MD Kusari S Lamshöft M Ngamga D Tane P Spiteller M Semi-synthesis of dihydrochalcone derivatives and their *in Vitro* antimicrobial activities Planta Med 2010 76 640 643 10.1055/s-0029-1240619 19937555

[B25] Lewis K Ausubel FM Prospects for plant derived antibacterials Nat Biotechnol 2006 24 1504 1507 10.1038/nbt1206-1504 17160050

[B26] Eloff JN Angeh I McGaw LJ A potentised leaf extract of *Melianthus comosus* has higher activity than six commercial products used against plant fungal pathogens S Afr J Bot 2007 73 286 287

[B27] Eloff JN Suleiman M Naidoo V A crude extract of *Loxostylus alata* is as effective in treating aspergillosis in poultry as a commercial drug Planta Med 2010 76 405

[B28] Tanamatayarat P Limtrakul P Chunsakaow S Duangrat C Screening of some Rubiaceous plants for cytotoxic activity against *Cervix Carcinoma* (KB-3-1) Cell Line Thai J Pharm Sci 2003 27 167 172

[B29] Caamal-Fuentes E Torres-Tapia LW Simá-Polanco P Peraza-Sánchez SR Moo-Puc R Screening of plants used in Mayan traditional medicine to treat cancer-like symptoms J Ethnopharmacol 2011 135 719 724 10.1016/j.jep.2011.04.004 21501677

[B30] Eloff JN A proposal on expressing the antibacterial activity of plant extracts - a small first step in applying scientific knowledge to rural primary health care in South Africa S Afr J Sci 2000 96 116 118

[B31] Eloff JN Famakin JO Katerere DRP *Combretum woodii* (Combretaceae) leaf extracts have high activity against Gram-negative and Gram-positive bacteria Afr J Biotechnol 2005 4 1161 1166

